# Retraction: Involvement of NF-κB in the reversal of CYP3A down-regulation induced by sea buckthorn in BCG-induced rats

**DOI:** 10.1371/journal.pone.0354768

**Published:** 2026-07-28

**Authors:** The PLOS One Editors

After this article [1] was published, concerns were raised regarding Figs 1, 7, S2 and S3. Specifically,

- In Figs 1A and 1B, several regions appear similar both within and across panels.
- In Fig 7, the following appear similar to each other:
  - Lanes 4-6 in the iNOS panel of Fig 7B and lanes 3-5 of the CYP3A panel of Fig 7C, when levels are adjusted and the panel flipped horizontally.
  - Lane 2 of the iNOS panel of Fig 7B and lane 3 of the CYP3A panel of Fig 7C, when levels are adjusted.
  - Lanes 1 and 3 of the GAPDH panel of Fig 7B (in the original figure, “GAPDH” is misspelled as “GADPH”).
  - Lanes 2 and 3 of the GAPDH panel of Fig 7C.
- In S2 Fig, the bands underlying both panels in Fig 7B appear discontinuous with the adjacent background.
- In S3 Fig, the bands underlying the CYP3A panel in Fig 7C appear discontinuous with the adjacent background.

The authors stated that Figs 1A and 1B are incorrect, and that local adjustments were made to these panels to improve the appearance of the images. They further stated that Fig 7B was incorrectly positioned, and that after electrophoresis and membrane transfer, the membrane was cut, and the target and reference proteins incubated and imaged separately. The underlying data provided did not resolve the above concerns.

In light of the above concerns that question the reliability and integrity of the reported results and conclusions, the *PLOS One* Editors retract this article.

FL, TW, XL, JJ, QL, and YX did not agree with retraction and stand by the article’s findings.
